# Supplementary material for: Dynamics of the microbiota in patients with Clostridioides difficile: Recurrence, treatment, sex, and immunosuppression
Source: PLoS Pathog. 2026 Apr 6;22(4):e1014063. doi: 10.1371/journal.ppat.1014063 (PMC13086424; doi:10.1371/journal.ppat.1014063)
Supplement: S1 Table — FDX: Fidaxomicin, VCN: Vancomycin, BZL: Bezlotuzumad. (DOCX) [file ppat.1014063.s001.docx]

**S1 Table.** Characteristics and Management of *Clostridioides difficile* Recurrence Episodes. FDX: Fidaxomicin, VCN: Vancomycin, BZL: Bezlotuzumad

|  | **1st Recurrence (N=32)** | **2nd Recurrence (N=11)** | **3rd Recurrence (N=3)** | **p-value** |
| --- | --- | --- | --- | --- |
| **Setting of CDI acquisition** |  |  |  | 0.089 |
| Community-acquired, %, (N) | 37.5 (12/32) | 72.7% (8/11) | 66.7% (2/3) |  |
| Hospital-acquired (>72h), %, (N) | 15.6 (5/32) | 0.0% (0/11) | 0.0% (0/3) |  |
| Healthcare-associated, %, (N) | 46.9 (15/32) | 27.3% (3/11) | 33.3% (1/3) |  |
| **Hospitalization (Yes) %, (N)** | 50.0 (16/32) | 18.2% (2/11) | 0.0% (0/3) | **0.046** |
| **New use of antibiotics (Yes)** %, (N) | 37.5 (12/32) | 27.3% (3/11) | 33.3% (1/3) | 0.701 |
| **Severity: Severe colitis** %, (N) | 21.9 (7/32) | 9.1% (1/11) | 66.7% (2/3) | 0.126 |
| **Treatment** |  |  |  | **0.024** |
| FDX (any scheme) %, (N) | 59.4 (19/32) | 18.2% (2/11) | 33.3% (1/3) |  |
| FDX + BLZ %, (N) | 6.3 (2/32) | 0.0% (0/11) | 0.0% (0/3) |  |
| VNC (any scheme) %, (N) | 9.4 (3/32) | 36.4% (4/11) | 33.3% (1/3) |  |
| VNC + BLZ %, (N) | 12.5 (4/32) | 36.4% (4/11) | 0.0% (0/3) |  |
| No treatment %, (N) | 6.3 (2/32) | 0.0% (0/11) | 0.0% (0/3) |  |
| Other%, (N) | 6.3 (2/32) | 9.1% (1/11) | 33.3% (1/3) |  |
