# Supplementary material for: Dynamics of the microbiota in patients with Clostridioides difficile: Recurrence, treatment, sex, and immunosuppression
Source: PLoS Pathog. 2026 Apr 6;22(4):e1014063. doi: 10.1371/journal.ppat.1014063 (PMC13086424; doi:10.1371/journal.ppat.1014063)
Supplement: S2 Table — (DOCX) [file ppat.1014063.s002.docx]

**S2 Table**. Recurrence rates according to the clinical and demographic variables analyzed.

|  |  | Rate of recurrence (%) | p-value |
| --- | --- | --- | --- |
| Sex | Male | 24.1 | 0.677 |
|  | Female | 21.2 |  |
| Age | <75 | 18.6 | 0.111 |
|  | ≥ 75 | 30.4 |  |
| Immunosuppression | No | 25.0 | 0.219 |
|  | Yes | 15.4 |  |
| Concomitant non-CDI antimicrobial therapy | No | 24.2 | 0.495 |
|  | Yes | 19.2 |  |
| Post-treatment non-CDI antimicrobial therapy | No | 18.3 | 0.324 |
|  | Yes | 25.3 |  |
| ICD treatment | VNC | 25.0 | 0.511 |
|  | FDX | 15.2 |  |
|  | VNC-BZL | 22.7 |  |
