# Supplementary material for: Dynamics of the microbiota in patients with Clostridioides difficile: Recurrence, treatment, sex, and immunosuppression
Source: PLoS Pathog. 2026 Apr 6;22(4):e1014063. doi: 10.1371/journal.ppat.1014063 (PMC13086424; doi:10.1371/journal.ppat.1014063)
Supplement: S3 Table — (DOCX) [file ppat.1014063.s003.docx]

**S3 Table**. Percentages according to sex, age and immunosuppression according to concomitant and post-treatment antimicrobial therapy of CDI, and treatment of CDI.

|  | **Sex** | | |  | **Age** | | |  | **Immunosuppression** | | |
| --- | --- | --- | --- | --- | --- | --- | --- | --- | --- | --- | --- |
|  | Male | Female | *p* |  | <75 | ≥ 75 | *p* |  | No | Yes | *p* |
| Concomitant antibiotics | 34.5 | 37.6 | 0.70 |  | 37.1 | 34.8 | 0.79 |  | 34.6 | 41.0 | 0.48 |
| Post-CDI antibiotics | 58.6 | 57.6 | 0.91 |  | 52.6 | 69.6 | 0.054 |  | 52.9 | 71.8 | 0.041 |
| VNC | 67.2 | 57.6 | 0.38 |  | 60.8 | 63.0 | 0.87 |  | 71.2 | 35.9 | 0.001 |
| FDX | 17.2 | 27.1 |  |  | 22.7 | 23.9 |  |  | 18.3 | 35.9 |  |
| VNC-BZL | 15.5 | 15.3 |  |  | 16.5 | 13.0 |  |  | 10.6 | 28.2 |  |

BLZ: bezlotuzumad; CDI: *Clostridioides difficile* infection; FDX: fidaxomicin; VNC: vancomycin
