# Supplementary material for: Dynamics of the microbiota in patients with Clostridioides difficile: Recurrence, treatment, sex, and immunosuppression
Source: PLoS Pathog. 2026 Apr 6;22(4):e1014063. doi: 10.1371/journal.ppat.1014063 (PMC13086424; doi:10.1371/journal.ppat.1014063)
Supplement: S4 Table — (DOCX) [file ppat.1014063.s004.docx]

|  | | **Diagnosis** | | | | **End-of-treatment** | | | | | | **8 weeks post-treatment/rec samples** | | | |
| --- | --- | --- | --- | --- | --- | --- | --- | --- | --- | --- | --- | --- | --- | --- | --- |
|  |  | **Shannon** | **p** | **ASVs** | **p** | **Shannon** | | **p** | **ASVs** | | **p** | **Shannon** | **p** | **ASVs** | **p** |
| **Patients without recurrence (n=111)** | | | | | | | | | | | | | | | |
| Male (n=44) | | 3.07±0.08 | 0.53 | 107.09±8.42 | 0.71 | 2.63±0.10 | 0.15 | | | 73.77±5.81 | 0.082 | 3.53±0.09 | 0.12 | 159.97±11.36 | 0.056 |
| Female (n=67) | | 3.15±0.07 |  | 111.04±6.52 |  | 2.43±0.08 |  |  |  | 60.98±4.34 |  | 3.31±0.08 |  | 134.61±8.48 |  |
| Age < 75 years (n=78) | | 3.08±0.06 | 0.27 | 105.02±6.17 | 0.18 | 2.47±0.08 | 0.30 | | | 66.55±4.45 | 0.83 | 3.40±0.07 | 0.77 | 146.02±8.00 | 0.84 |
| Age ≥ 75 (n=33) | | 3.21±0.09 |  | 120.00±9.14 |  | 2.62±0.09 |  |  |  | 64.87±5.55 |  | 3.37±0.11 |  | 141.45±13.57 |  |
| Immunocompetent (n=78) | | 3.16±0.06 | 0.63 | 112.94±6.57 | 0.30 | 2.47±0.08 | 0.39 | | | 64.93±4.41 | 0.63 | 3.45±0.08 | 0.14 | 152.50±8.71 | 0.051 |
| Immunosuppressed  (n=33) | | 3.02±0.09 |  | 101.27±7.55 |  | 2.60±0.10 |  |  |  | 68.69±5.71 |  | 3.26±0.10 |  | 126.15±10.08 |  |
| CDI treatment | VNC (n=66) | 3.13±0.08 | 0.92 | 113.37±7.97 | 0.61 | 2.40±0.08 | <0.001 | | | 61.09±4.59 | 0.053 | 3.49±0.07 | 0.54 | 149.16±8.19 | 0.17 |
|  | FDX (n=28) | 3.13±0.08 |  | 101.28±5.69 |  | 2.95±0.11 |  |  |  | 80.71±6.84 |  | 3.28±0.14 |  | 144.75±16.94 |  |
|  | VNC-BLZ (n=17) | 3.06±0.11 |  | 107.82 |  | 2.23±0.16 |  |  |  | 61.17±8.10 |  | 3.21±0.21 |  | 127.05 |  |
| **Patients with recurrence (n=20)*** **^Only patients with paired samples have been included in this analysis.^** | | | | | | | | | | | | | | | |
| Male (n=6) | | 3.25±0.22 | 0.46 | 137.83±29.48 | 0.16 | 3.01±0.28 | 0.17 | | | 113.66±44.89 | 0.043 | 3.51±0.23 | 0.20 | 150.50±24.99 | 0.034 |
| Female (n=14) | | 3.06±0.12 |  | 89.28±8.02 |  | 2.45±0.25 |  |  |  | 56.64±13.67 |  | 3.13±0.14 |  | 104.71±1.94 |  |
| Age < 75 years (n=9) | | 3.15±0.19 | 0.78 | 106.55±19.63 | 0.83 | 2.65±0.39 | 0.90 | | | 96.66±35.29 | 0.28 | 3.40±0.14 | 0.25 | 135.33±17.95 | 0.13 |
| Age ≥ 75 years (n=11) | | 3.08±0.12 |  | 101.63±13.41 |  | 2.60±0.19 |  |  |  | 55.00±9.47 |  | 3.11±0.19 |  | 104.63±10.08 |  |
| Immunocompetent (n=16) | | 3.04±0.11 | 0.20 | 96.00±9.99 | 0.17 | 2.64±0.16 | 0.92 | | | 60.25±11.59 | 0.41 | 3.24±0.14 | 0.98 | 115.00±7.84 | 0.72 |
| Immunosuppressed (n=14) | | 3.40±0.30 |  | 135.25±39.56 |  | 2.55±0.87 |  |  |  | 127.75±71.48 |  | 3.24±0.33 |  | 132.25±44.07 |  |
| CDI | VNC (n=14) | 3.06±0.10 | 0.71 | 100.78±10.70 | 0.74 | 2.63±0.18 | 0.030 | | | 63.28±13.92 | 0.19 | 3.17±0.16 | 0.34 | 109.42±8.68 | 0.31 |
| treatment | FDXc(n=4) | 3.30±0.41 |  | 121.00±45.10 |  | 3.26±0.39 |  | | | 132.00±66.78 |  | 3.61±0.20 |  | 149.50±39.56 |  |
|  | VNC-BLZ(n=2) | 3.11±0.20 |  | 91.00±20.00 |  | 1.27±1.10 |  |  |  | 30.50±15.50 |  | 3.00±0.27 |  | 119.50±27.50 |  |

**S4 Table**. Mean ± se values of α diversity according to study time points and explanatory variables and pvalue for comparison among groups

ASV: amplicon variant sequences; BLZ: bezlotuzumad; CDI: *Clostridioides difficile* infection; FDX: fidaxomicin; VNC: vancomycin., VNC-BLZ: vancomycin-bezlotuzumad
