# Supplementary material for: Dynamics of the microbiota in patients with Clostridioides difficile: Recurrence, treatment, sex, and immunosuppression
Source: PLoS Pathog. 2026 Apr 6;22(4):e1014063. doi: 10.1371/journal.ppat.1014063 (PMC13086424; doi:10.1371/journal.ppat.1014063)
Supplement: Attachment — Submitted filename: Review for PLOS Pathogens.pdf [file ppat.1014063.s008.pdf]

C. difficile infection (CDI) is known to be associated with loss of gut microbiome diversity. This manuscript describes longitudinal microbiome changes in a cohort of patients treated with different antibiotic regimens for CDI. Microbiome dynamics are shown to be different in patients treated with vancomycin vs fidaxomicin, as well as in recurrence. The authors also invoke a role for gender and immunocompetence status in microbiome trajectories.

Major Comments:

1. The main conclusions largely confirm previously published reports that are unfortunately not considered in this manuscript. The authors need to assess their results in the light of these other studies, noting the concurrence of results. There is always value in validation of results with different cohorts.

   Specifically, regarding three of the four highlights listed (lines 44-53), the authors might consider the following manuscripts:

   i. Okhuysen PC, Ramesh MS, Louie T, Kiknadze N, Torre-Cisneros J, de Oliveira CM, Van Steenkiste C, Stychneuskaya A, Garey KW, Garcia-Diaz J, Li J, Duperchy E, Chang BY, Sukbuntherng J, Montoya JG, Styles L, Clow F, James D, Dubberke ER, Wilcox M. A randomized, double-blind, Phase 3 Safety and Efficacy study of ridinilazole versus vancomycin for treatment of *Clostridioides difficile* infection: clinical outcomes with microbiome and metabolome correlates of response. Clin Infect Dis. 2024 Jun 14;78(6):1462-1472. doi: 10.1093/cid/ciad792. PMID: 38305378; PMCID: PMC11175683.

   ii. Seekatz, A.M., Rao, K., Santhosh, K. *et al.* Dynamics of the fecal microbiome in patients with recurrent and nonrecurrent *Clostridium difficile* infection. *Genome Med* **8**, 47 (2016). https://doi.org/10.1186/s13073-016-0298-8

   iii. Louie TJ, Cannon K, Byrne B, Emery J, Ward L, Eyben M, Krulicki W. Fidaxomicin preserves the intestinal microbiome during and after treatment of Clostridium difficile infection (CDI) and reduces both toxin reexpression and recurrence of CDI. Clin Infect Dis. 2012 Aug;55 Suppl 2(Suppl 2):S132-42. doi: 10.1093/cid/cis338. PMID: 22752862; PMCID: PMC3388020

2. As noted in the fourth highlight (lines 52-53), the authors determined that recovery of microbiota diversity at 8 weeks is slower in women and immunosuppressed patients. How many of the immunosuppressed individuals were women? It might be more helpful to only consider immunocompetent men and women when evaluating gender influences on microbiome dynamics.

Specific other comments:

1. Line numbering ends towards the end of Results section. There are also no page numbers.

2. A single table providing demographics of the patient cohort, combining the relevant data from Table 1 and S1 would be valuable for ease of understanding by the reader.

3. Much of the microbiome-related data in Table 1 is more readily observed in Figure 2. Perhaps these data could be moved to supplementary data.

4. Since this paper is focused on antibiotic effects, it would be meaningful to better describe the treatment regimens (dose, length of treatment). It would help to have time of recurrence/relapse also presented in the tables.

5. Bezlotoxumab is referred to variously as bezlotuzumab/bezlotuzumad and should be referred to correctly. Some comment should also be included on whether bezlotoxumab treatment made any difference to microbiome outcome.

6. Please define Dx, and rec when introducing for the first time (Lines 121-122).

7. Maintain consistency with regard to use of H-index/Shannon diversity index in text and figure (line 139, Figures 1 and 2).
