# Supplementary material for: Dynamics of the microbiota in patients with Clostridioides difficile: Recurrence, treatment, sex, and immunosuppression
Source: PLoS Pathog. 2026 Apr 6;22(4):e1014063. doi: 10.1371/journal.ppat.1014063 (PMC13086424; doi:10.1371/journal.ppat.1014063)
Supplement: Attachment — Submitted filename: Response to reviewers.docx [file ppat.1014063.s009.docx]

**Dear editor,**

The authors would like to extend their gratitude to both the editor and reviewer for their assistance in reviewing this study. We greatly value their support. The reviewer’s comments have been addressed and incorporated into the manuscript as outlined below:

**Manuscript ID: PPATHOGENS-D-25-02898**

**Title: "** **Dynamics of the microbiota in patients with *Clostridioides difficile*: recurrence, treatment, sex, and immunosuppression.”**

**Reviewers’ Comments**

**Part I – Summary**

**Reviewer #1:**

The authors thank the reviewer for their careful evaluation of the manuscript and their constructive feedback.

1. The main conclusions largely confirm previously published reports that are unfortunately not considered in this manuscript. The authors need to assess their results in the light of these other studies, noting the concurrence of results. There is always value in validation of results with different cohorts.
Specifically, regarding three of the four highlights listed (lines 44-53), the authors might consider the following manuscripts:

i. Okhuysen PC, Ramesh MS, Louie T, Kiknadze N, Torre-Cisneros J, de Oliveira CM, Van Steenkiste C, Stychneuskaya A, Garey KW, Garcia-Diaz J, Li J, Duperchy E, Chang BY, Sukbuntherng J, Montoya JG, Styles L, Clow F, James D, Dubberke ER, Wilcox M. A randomized, double-blind, Phase 3 Safety and Efficacy study of ridinilazole versus vancomycin for treatment of Clostridioides difficile infection: clinical outcomes with microbiome and metabolome correlates of response. Clin Infect Dis. 2024 Jun 14;78(6):1462-1472. doi: 10.1093/cid/ciad792. PMID: 38305378; PMCID: PMC11175683.

ii. Seekatz, A.M., Rao, K., Santhosh, K. et al. Dynamics of the fecal microbiome in patients with recurrent and nonrecurrent Clostridium difficile infection. Genome Med 8, 47 (2016). <https://doi.org/10.1186/s13073-016-0298-8>

iii. Louie TJ, Cannon K, Byrne B, Emery J, Ward L, Eyben M, Krulicki W. Fidaxomicin preserves the intestinal microbiome during and after treatment of Clostridium difficile infection (CDI) and reduces both toxin reexpression and recurrence of CDI. Clin Infect Dis. 2012 Aug;55 Suppl 2(Suppl 2):S132-42. doi: 10.1093/cid/cis338. PMID: 22752862; PMCID: PMC3388020.

Thank you for bringing these relevant references to our attention. We have now incorporated all of them into the Discussion section and revised the text accordingly to better contextualize our findings within the existing literature.

*To date, few published studies have analyzed the microbiota longitudinally with such a long follow-up and in such a large sample of patients as in our study. Nevertheless, our results are in line with previous reports. In particular, Seekatz et al.* [11] *showed in a smaller cohort with intra-individual comparisons that patients who experienced recurrence were less likely to recover microbiota diversity, and that recurrence was associated with persistently reduced diversity throughout the CDI episode* [12]*. These findings support our observation that impaired microbiota recovery is a key determinant of recurrence.~~However, it has been reported that the recurrence has been linked to less diversity throughout the CDI episode~~* ~~[12]~~ *~~.~~* (Lines 382-389)

*These findings support the role of FDX in preserving microbiota integrity, which is crucial for preventing recurrence, and are agree with previous studies showing that VNC causes greater dysbiosis in the microbiome in patients with CDI* [20]*, while decreasing the proportion of some genera associated with a healthy microbiome, such as Bacteroides* [21]*. In this regard, members of the phylum Bacteroidetes* appear to be critical to maintaining gut homeostasis. For all these reasons, FDX *has been consistently identified as a protective agent against CDI recurrence* [16,22,23]*. ~~Among protective microbiota features, members of the phylum Bacteroidetes appear to be critical to maintaining gut homeostasis.~~ Our findings are consistent with prior reports showing that FDX has a limited deleterious effect on this phylum compared to VNC* [20,21,24]*.* (Lines 410-417)

2. As noted in the fourth highlight (lines 52-53), the authors determined that recovery of microbiota diversity at 8 weeks is slower in women and immunosuppressed patients. How many of the immunosuppressed individuals were women? It might be more helpful to only consider immunocompetent men and women when evaluating gender influences on microbiome dynamics.

Thank you for your feedback. Following the recommendation of the reviewer, the analysis was repeated using only the immunocompetent group. The results were the same as those obtained in the general population, which reinforces the findings. The following paragraph has been added to the 'Results' section:

*To evaluate the effect of sex on microbiome recovery minimizing the potential confounding effect of immunosuppression, the same analysis was conducted including only immunocompetent patients. In this subgroup, 39.75% of patients were male, a proportion comparable to that of the overall study population. Consistent with our primary analysis, microbiota diversity recovery at eight weeks differed by sex. In women, diversity at eight weeks (ASVs = 141.73, H = 3.33) remained similar to that observed in the CDI diagnosis sample (ASVs = 113.55, H = 3.18) (ASVs: p = 0.30, H: p = 0.86). However, men exhibited a significantly higher diversity at eight weeks (ASVs = 169.48, H = 3.64) compared with diagnostic sample (ASVs = 112.74, H = 3.13) (ASVs: p = 0.002, H: p = 0.002).* (Lines 252-261)

**Reviewer #2:**

The authors sincerely appreciate the reviewer’s thorough review and valuable suggestions, which helped to strengthen this manuscript.

This article has focused on studying how the treatment of *C. difficile* diarrhoea affects the gut microbiota and its relationship with recurrence. Its strengths are that it has a considerable number of patients, the statistical analysis is well done, and significant results are found for the different treatments. The limitations are that the characteristics of the patients have been poorly defined, and this could be a significant handicap.

Thank you for your comment. In order to assess the limitation indicated by the reviewer, an initial descriptive table has been added with the clinical characteristics of the patients studied, as detailed in the Part II.

**Reviewer #3**

Review of Ventero et al., “Dynamics of the microbiota in patients with Clostridioides difficile: recurrence, treatment, sex, and immunosuppression”
This manuscript investigates microbiota dynamics in patients with *Clostridioides difficile* infection (CDI), focusing on recurrence, treatment modality, sex, age, and immunosuppression. The study includes 143 patients and is generally well written. The authors report that patients who experience recurrent CDI exhibit lower microbial diversity compared to those who do not relapse, and that fidaxomicin better preserves gut microbiota relative to vancomycin, either alone or combined with bezlotoxumab. These findings are broadly consistent with previously published studies and therefore largely confirmatory.

The manuscript addresses a clinically relevant topic and includes a substantial cohort. However, the analytical approach requires refinement to ensure robust and interpretable results.

We appreciate your response. The additional analyses requested by the reviewer have been carried out, as detailed in Part II.

**Part II Major Issues: Key Experiments Required for Acceptance**

**Reviewer #1:**

(No Response)

**Reviewer #2:**

- It is necessary to describe the characteristics of the patients; it has not been specified whether the CDI episode is primary or whether they have had any recurrences. Reference is made to antibiotic use during CDI treatment, but it might be interesting to include previous exposure.
- There is no explanation of how the treatment is chosen, why some patients receive fidaxomicin and others vancomycin, only that vancomycin is less commonly used in immunocompromised patients.
- It would be interesting to describe the changes in recurrences and after treatment of recurrence.

The authors agree with the reviewer's comments and appreciate his timely observations, which are discussed separately in the following paragraphs.

- It is necessary to describe the characteristics of the patients; it has not been specified whether the CDI episode is primary or whether they have had any recurrences. Reference is made to antibiotic use during CDI treatment, but it might be interesting to include previous exposure.

The results section, now includes a descriptive table (Table 1) of the clinical characteristics of the study population, as well as a paragraph summarising this information.

*Most episodes were healthcare-associated or hospital-acquired (40.6%), and nearly three quarters of patients required hospitalization. Comorbidity burden was substantial, with a median Charlson Comorbidity Index of 4 (IQR 2–6). Prior healthcare exposure was frequent, including recent hospitalization and antibiotic use in the preceding three months in over 90% of cases. At the time of CDI diagnosis, 44.7% of patients were receiving active antibiotic therapy, and more than half had chronic proton pump inhibitor use. Severe disease or a complicated clinical course was observed in 23.1% of cases, with low rates of sepsis and ICU admission. Recurrence occurred in approximately one fifth of patients (22.4%) (Table 1).* (Lines 131-140)

- There is no explanation of how the treatment is chosen, why some patients receive fidaxomicin and others vancomycin, only that vancomycin is less commonly used in immunocompromised patients.

Treatment assignation was carried out in accordance with current guidelines (IDSA and ESCMID) for the management of CDI, and by specialised PROA teams that establish treatment recommendations in the different hospitals participating in the study.

The authors added a description of how the treatment was chosen in the material and methods section:

*Treatment for C. difficile infection was selected in accordance with current ESCMID [29] and IDSA [30] guidelines, and within a structured, multidisciplinary clinical pathway for systematic case assessment and treatment indication [31]. VNC or FDX was used for initial episodes based on recurrence risk, and FDX or VNC plus BZL for first recurrences. (Lines 471-475)*

- It would be interesting to describe the changes in recurrences and after treatment of recurrence.

We thank the reviewer for this suggestion. Unfortunately, microbiome analyses were not performed in patients with recurrent CDI, and therefore changes in microbiota composition after recurrence treatment cannot be assessed. However, to address this comment, we have added a new supplementary table (S3) describing the demographic characteristics, clinical features, treatment regimens, and outcomes of all patients who developed recurrence. In the revised manuscript, we now highlight the most relevant findings, including the setting of acquisition, severity, antibiotic exposure prior to recurrence, and treatment strategies used for recurrent episodes.

*Out of the total cohort, 32 (22.4%) patients experienced a first recurrence of Clostridioides difficile infection (rCDI), of whom 11 progressed to a second recurrence, and 3 subsequently developed a third episode. The first recurrence was predominantly associated with healthcare-related settings (46.9%), subsequent episodes occurred mainly in community-dwelling patients (p=0.089). A significant reduction in the requirement for hospitalization was observed as the number of recurrences increased, dropping from 50.0% in the first episode to 18.2% in the second (p=0.046). Furthermore, therapeutic strategies evolved significantly across episodes (p=0.024); although FDX monotherapy was the preferred choice for the first recurrence (59.4%), management of the second recurrence shifted toward the use of VNC-based regimens and the intensified use of BZL (36.4%) (S1).* (Lines 144-155)

**Reviewer #3:**

•Analytical Concerns:

o There is no indication that p-values were adjusted for multiple testing. Given the number of comparisons, this is essential to reduce false positives.

The authors thank the reviewer for this comment. In the Material and Methods section, it has been clarified that p-values were adjusted for multiple comparisons, using the holm method for longitudinal comparisons of alpha diversity, and FDR (internal false discovery rate) correction for ANCOM-BC analysis. In addition, the paragraph describing the parameters and corrections used in the ANCOM-BC analysis has been expanded and clarified.

*The genera differentially represented were determined using ANCOM-BC (4.1.0), which accounts for the compositional nature of microbiome data and applies an internal correction for multiple comparisons bases on the false discovery rate (FDR). Genera with FDR‑adjusted q values < 0.05 and absolute log2 fold (|log2FC|) > 2 were considered statistically significant. The robustness of the results was evaluated using pseudcount sensitivity analysis and only accepted the genera when this parameter was true. ~~change and defined as such when the adjusted p value in the ANCOM analysis was less than 0.05, the log fold change was greater than -2/2, and the pseudocount sensitivity was true.~~* (Lines 492-501)

*For paired comparisons across time points, paired t-test or repeated measures ANOVA were used for normally distributed data, while the Wilcoxon signed-rank test or Friedman’s test for non-normally distributed data. For comparisons between independent groups with normally distributed data, Welch’s t-test was used.* ***P-values were adjusted for multiple testing using the holm method.*** (Lines 510-512)

o The manuscript states that t-tests were used for parametric data but does not specify which type. Welch’s t-test is generally recommended when sample sizes and variances differ between groups.

This commentary has been incorporated together with the previous one, and it has been indicated that the t-test used for these comparisons was Welch's test, since, as the reviewer points out, this test is appropriate and widely accepted for this type of analysis.

*For paired comparisons across time points, paired t-test or repeated measures ANOVA were used for normally distributed data, while the Wilcoxon signed-rank test or Friedman’s test for non-normally distributed data.* ***For comparisons between independent groups with normally distributed data, Welch’s t-test was used.*** *P-values were adjusted for multiple testing using the holm method.* (Lines 510-512)

o Sampling timepoints are not matched between outcome groups. While relapse timing is inherently unpredictable, comparing fixed post-treatment intervals in non-recurrent patients to variable relapse timepoints introduces bias. Time-adjusted mixed-effects models for Shannon diversity and ASV counts would provide a more robust analysis.

o The impact of treatment (vancomycin vs. fidaxomicin vs. vancomycin + bezlotoxumab) on alpha diversity is not adjusted for key confounders and does not account for repeated measures. Mixed-effects models are standard for longitudinal microbiome data and should be employed.

The authors thank the reviewer for highlighting these two points. As both relate to linear models, they have been grouped together to be addressed jointly.

Firstly, to minimise the bias introduced by the different sampling times, a new continuous time variable was included, recording the days on which each sample was collected. This variable was included in a time-adjusted longitudinal mixed-effects model to assess the effect of recurrence over time, reducing the bias indicated by the reviewer. The results were similar to the previous ones. The linear model indicated that patients without recurrence recover 0.672 ASVs more per day than patients with it, in whom this variable did not increase but even tends to decreased (-0.06, pv=0.021) (**Figure 1C**). Similarly, although not as markedly, the Shannon index stabilised per day in patients with recurrence, while in patients without recurrence it increased by 0.01 units per day (p=0.088). This indicates that ASVs show greater sensitivity than the Shannon index in our population for detecting longitudinal changes in the microbiome.

This detailed explanation has been added to the Results section, along with a new figure showing the linear model generated.

To evaluate the effect of recurrence on microbiota diversity over time using a longitudinal approach, time was included as a continuous variable (defined as the number of days from diagnosis to sample collection). This variable was incorporated into a time-adjusted longitudinal mixed-effects model, yielding results consistent with previous analyses. The linear model showed that patients without recurrence recovered, on average, 0.672 additional ASVs per day, whereas in patients with recurrence this variable did not increase and instead showed a slight decreasing trend (−0.06 ASVs per day, p = 0.021) **(Figure 1C, S6,S7**). Similarly, although less markedly, the Shannon diversity index remained stable over time in patients with recurrence, while it increased by 0.01 units per day in patients without recurrence (p = 0.088). (Lines 193-203)

With regard to the second point, and in order to integrate all the results presented in the Results section into a single model, a longitudinal linear model was constructed including all variables that were significant in the univariate analyses of microbiome dynamics (recurrence, CDI treatment, sex, and immunosuppression). However, some subgroups had zero observations (e.g., immunocompromised men with recurrence treated with fidaxomicin), which compromised the robustness of the model. Therefore, to obtain a more reliable model, a final model including recurrence, sex, and CDI treatment was generated. This model confirmed all previous findings.

In addition, linear models were also performed in the non-recurrent patient population, including CDI treatment, sex, and immunosuppression as variables. Similar results were obtained, with p-values close to the threshold for statistical significance.

A paragraph describing these models has been added at the end of the Results section, and the results are shown in Figure 3

*“In order to assess the results globally, all variables that showed a significant association in the univariate analysis (recurrence, CDI treatment, sex, and immunosuppression) were included in a longitudinal linear mixed-effects model. In this model, several subgroups with zero observations were identified, which compromised the robustness of the estimates. Therefore, a final model including recurrence, CDI treatment, and sex was generated. The results of this model were consistent with those previously reported.*

*For both, observed ASVs and the Shannon diversity index, a significant effect of time on microbiome recovery was observed depending on recurrence status. Patients with recurrence exhibited a significantly lower recovery of microbiota diversity over time (ASVs: β = −1.936 [−3.147 to −0.725], p = 0.002; Shannon: β = −0.031 [−0.056 to −0.005], p = 0.018). Sex was also associated with differential recovery of observed ASVs over time, with women showing a significantly lower recovery rate (ASVs: β = −0.496 [−0.939 to −0.053], p = 0.028) (****Figure 3A****).With regard to CDI treatment, differences were observed in the longitudinal evolution of the Shannon diversity index between patients treated with vancomycin and fidaxomicin (β = −0.016 [−0.030 to −0.001], p = 0.032), with the latter group showing less variation in this index over time (****Figure 3B****).*

*These models were also repeated in the subgroup of patients without recurrence, including immunosuppression as a covariate. The same trends were observed, although statistical significance remained close to the threshold. For ASV richness, the p-value for the interaction between time and sex was 0.077, and for the Shannon index the p-value for the interaction between time and treatment was 0.078. The results of all fitted models are provided in the supplementary material (****S6, S7****).” (Lines 264-293)*

o Figure 1 relies on observed ASVs and the Shannon index. Observed ASVs are highly sensitive to sequencing depth, yet normalization procedures and sensitivity analyses (e.g., rarefaction) are not reported. Apparent recovery in Figure 1 may reflect differences in sequencing depth rather than biological change.

We completely agree with the reviewer's comment. Prior to the diversity analysis, rarefaction curves were generated and used to set a minimum depth threshold for all samples included in the analysis in order to avoid these biases. This information has now been added to the Material & Methods section and is indicated in the Results. Rarefaction curves are provided as Supplementary data S5.

*Material & Methods*

*Prior to diversity analyses, rarefaction curves were generated (****S4****) for the observed ASVs and for the H index in order to determine the sequencing depth threshold. A minimum sequencing depth of 10,000 reads was defined based on these curves, and only samples that reached this threshold were included in the analyses. (Lines 486-49)*

*Results:*

*All samples included in the diversity analyses met the minimum sequencing depth threshold defined by the rarefaction analysis (10,000 reads per sample,* ***S4****). (Lines 180-181).*

**Part III – Minor Issues: Editorial and Data Presentation Modifications**

**Reviewer #1:**

1. Line numbering ends towards the end of Results section. There are also no page numbers.

Line and page numbering has been included.

2. A single table providing demographics of the patient cohort, combining the relevant data from Table 1 and S1 would be valuable for ease of understanding by the reader.

We thank the reviewer for this suggestion. A new table (**Table 1**) summarizing the demographic characteristics of the cohort has been added to the manuscript. Supplementary Table S1 (now S2) has been retained to avoid excessive length of the main descriptive table.

3. Much of the microbiome-related data in Table 1 is more readily observed in Figure 2. Perhaps these data could be moved to supplementary data.

Thank you for your comment. Table 1 has been moved to the supplementary material (now S5). With this modification, suggested by the reviewer, the information is not repeated and the article is easier to read.

4. Since this paper is focused on antibiotic effects, it would be meaningful to better describe the treatment regimens (dose, length of treatment). It would help to have time of recurrence/relapse also presented in the tables.

This information has been added to Table 1 (new), which now includes, in addition to the demographic description of the cohort, variables related to the clinical course and treatment of the CDI episode. Furthermore, a supplementary table (S3) has been included to provide detailed information on recurrence episodes.

5. Bezlotoxumab is referred to variously as bezlotuzumab/bezlotuzumad and should be referred to correctly. Some comment should also be included on whether bezlotoxumab treatment made any difference to microbiome outcome.

The manuscript has been revised and now whenever Bezlotuzumab is referred to, it is spelled correctly.

With regard to the influence of bezlotuzumab on the microbiome, diversity analyses have shown no difference compared to patients treated with vancomycin alone. This assessment is included in the text:

*Patients treated with VNC-BZL exhibited a similar pattern during treatment, than patients treated with VNC.(Lines 219-220)*

*Samples from patients treated with VNZ-BLC showed an H-index comparable to that of patients treated with VNC (Lines 228-229)*

Taxonomic composition analyses also include assessments in this regard:

*Related to dynamics of microbiota composition, treatment with FDX did not alter the taxonomic composition of the microbiota, with similar relative abundance percentages maintained for the six phyla studied over the three time points. In patients treated with VNC, the taxonomic composition was modified in five out of the six phyla, and in the case of VNC-BZL, three of these six phyla were altered (Lines 327)*

*In contrast, no differentially represented genera were observed in patients treated with VNC-BZL versus VNC alone.(Lines 357-358)*

6. Please define Dx, and rec when introducing for the first time (Lines 121-122).

The abbreviations have now been correctly defined the first time they were used.

*and 84 samples from the 32 patients with recurrence (Diagnostic (Dx) n=32, End‑Treatment (ET) n=20 from patients responding well to the first round of treatment, and recurrence dx (rec), n=32). (Lines 163-165)*

7. Maintain consistency with regard to use of H-index/Shannon diversity index in text and figure (line 139, Figures 1 and 2).

This consistency has been reviewed throughout the entire manuscript and in the figures, and the first time the Shannon index is mentioned, it has been indicated that in the manuscript it is referred to as the H-index.

***In the overall cohort of patients,*** *a significant reduction in α diversity was observed after completion of treatment for CDI, as assessed by both the Shannon index (H-index). (Lines 186-187)*

**Reviewer #2:**

Figure 4, the label of Fusobacteria is wrong.

Now it’s ok.

Reviewer #3:

• The terms “H Index” and “Shannon Index” appear to be used interchangeably. It should be explicitly clarified that these refer to the same metric, or corrected if they do not.

The authors have revised and corrected this point, clearly describing what this index means the first time it is used. They have also ensured that consistency is maintained in this term throughout the manuscript and figures.

***In the overall cohort of patients,*** *a significant reduction in α diversity was observed after completion of treatment for CDI, as assessed by both the Shannon index (H-index). (Lines 186-187)*

• Figure legends.

o Abbreviations used in figures should be defined within the legends for reader clarity.

We added the definitions of the abbreviation in the figures.

***Figure 1****. Evolution of α diversity according to the time points studied. H-index and ASVs observed in the whole population (n=143) (****A****), and in recurrent patients (n=32) (****B****). Results of the longitudinal mixed-effects models generated, in green showed the estimated trend for non-recurrent patients, the red line illustrates the same for recurrent patients (****C****). Dx= sample from diagnostic, ET: sample from end-treatment, 8W: samples from 8 week post-treatment, rec: sample from recurrence diagnostic.(Lines 208-212)*

***Figure 5****. Evolution of taxonomic composition in non-relapsed patients according to CDI treatment (****A****) and sex (****B****). Dx= sample from diagnostic, ET: sample from end-treatment, 8W: samples from 8 week post-treatment, rec: sample from recurrence diagnostic. (Lines 337-340)*

o In Figure 3, the phrase “according to” could be replaced with “stratified by” for greater precision.

This phrase has been replaced.

***Figure 4****. Phyla with a relative abundance greater than 1% in the ET samples ~~according~~ ~~to~~ stratified by sex (****A****), CDI treatment (****B****) and recurrence (****C****). The x-axis represents the percentage of relative abundances~~. Percentages are shown in logarithmic scale for better visualization of the graph.~~* (Lines 319-322)

o The legend for Figure 3 states that “percentages are shown in logarithmic scale,” but the figure does not appear to use a logarithmic scale. This should be corrected.

The reviewer is absolutely right, and we apologise for this error. Finally, the figure was included with the percentages, and the conversion to logarithmic scale was not made to better reflect the relative abundance of each phylum. The figure and the figure caption has been corrected.

***Figure 4****. Phyla with a relative abundance greater than 1% in the ET samples ~~according~~ ~~to~~ stratified by sex (****A****), CDI treatment (****B****) and recurrence (****C****). The x-axis represents the percentage of relative abundances~~. Percentages are shown in logarithmic scale for better visualization of the graph.~~* (Lines 319-322)

• The manuscript appears to apply an arbitrary cutoff for displaying p-values. It would be preferable to either report all p-values or restrict reporting to those meeting a predefined significance threshold.

The authors have decided to show all p-values for greater transparency of the data reflected in the manuscript. All figures and tables have been modified in accordance with this decision.
